# The Effect of Ventilation, Age, and Asthmatic Condition on Ultrafine Particle Deposition in Children

**DOI:** 10.1155/2012/736290

**Published:** 2012-07-11

**Authors:** Hector A. Olvera, Daniel Perez, Juan W. Clague, Yung-Sung Cheng, Wen-Whai Li, Maria A. Amaya, Scott W. Burchiel, Marianne Berwick, Nicholas E. Pingitore

**Affiliations:** ^1^Center for Environmental Resource Management, University of Texas at El Paso, 500 W. University Avenue, El Paso, TX 79968, USA; ^2^Civil Engineering Department, University of Texas at El Paso, 500 W. University Avenue, El Paso, TX 79968, USA; ^3^Geological Sciences Department, University of Texas at El Paso, 500 W. University Avenue, El Paso, TX 79968, USA; ^4^Aerosol and Dosimetry Program, Lovelace Respiratory Research Institute, 2425 Ridgecrest Dr. SE, Albuquerque, NM 87108-5127, USA; ^5^School of Nursing, University of Texas at El Paso, 500 W. University Avenue, El Paso, TX 79968, USA; ^6^Center for Environmental Health Sciences, University of New Mexico, Los Lunas, NM 87131, USA

## Abstract

Ultrafine particles (UFPs) contribute to health risks associated with air pollution, especially respiratory disease in children. Nonetheless, experimental data on UFP deposition in asthmatic children has been minimal. In this study, the effect of ventilation, developing respiratory physiology, and asthmatic condition on the deposition efficiency of ultrafine particles in children was explored. Deposited fractions of UFP (10–200 nm) were determined in 9 asthmatic children, 8 nonasthmatic children, and 5 nonasthmatic adults. Deposition efficiencies in adults served as reference of fully developed respiratory physiologies. A validated deposition model was employed as an auxiliary tool to assess the independent effect of varying ventilation on deposition. Asthmatic conditions were confirmed via pre-and post-bronchodilator spirometry. Subjects were exposed to a hygroscopic aerosol with number geometric mean diameter of 27–31 nm, geometric standard deviation of 1.8–2.0, and concentration of 1.2 × 10^6^ particles cm^−3^. Exposure was through a silicone mouthpiece. Total deposited fraction (TDF) and normalized deposition rate were 50% and 32% higher in children than in adults. Accounting for tidal volume and age variation, TDF was 21% higher in asthmatic than in non-asthmatic children. The higher health risks of air pollution exposure observed in children and asthmatics might be augmented by their susceptibility to higher dosages of UFP.

## 1. Introduction

Particles smaller than 100 nm, due to their size, can elude human defense mechanisms, penetrate deep into the body, reach the bloodstream, and accumulate in sensitive target sites such as bone marrow, lymph nodes, spleen, heart, brain, and the central nervous system [1–9]. The distinctive translocation properties of nanoparticles have prompted their application as drug carrying vectors and in early detection, diagnosis, and treatment of diseases [7, 10–19]. Unfortunately, such translocation properties might also explain why ultrafine particles (UFP) significantly contribute to the elevated health risks associated with urban air pollution [3, 20–22]. In particular, UFPs have been shown to impact the cardiovascular, pulmonary, and central nervous systems, especially in children, the elderly, and those with respiratory diseases [5, 20–26]. Exposure to UFPs has also been linked to pulmonary inflammation and increased susceptibility to respiratory infections as well as increased risk of cancer, chronic obstructive pulmonary diseases, and exacerbation of asthma [27–38]. 

Despite extensive research on the health effects of air pollution, the fundamental mechanisms by which UFP could induce disease remain elusive. Further research (e.g., absorption, biopersistence, carcinogenicity, translocation to other tissues or organs, etc.) is necessary to support a comprehensive assessment of the risks associated with human inhalation exposure to UFP. Advances in the epidemiology, toxicology, and pharmacology of nanoparticles hinge on the ability to accurately determine dose-based susceptibility associated with inhalation exposure. As prevalence of asthma and other respiratory illnesses remains high among children, understanding their underlying susceptibility to air pollution is urgent [39–43]. In asthmatics, greater UFP deposition might be induced by enhanced diffusional mechanisms caused by airway obstruction and increased alveolar volumes. In children, however, developing respiratory physiology and changes in breathing patterns could further induce deposition variability [44–46]. In adults, asthmatic conditions have been observed to significantly increase UFP deposition [47]. In children, the effect of asthmatic conditions on deposited fraction of ultrafine particles remains undetermined. The objective of this study was to explore the effect of ventilation, developing respiratory physiology, and asthmatic condition on the deposition efficiency of poly-dispersed hygroscopic ultrafine particles in children. Deposition efficiencies of healthy adults were determined to serve as reference of fully developed respiratory physiologies. The International Commission on Radiological Protection deposition model [48] was employed as an auxiliary tool to assess the independent effect of ventilation on deposition efficiency.

## 2. Methods

### 2.1. Subjects 

The Institutional Review Board for the protection of human subjects participating in research at the University of Texas at El Paso approved the research protocol (no. 93915). Informed written consent forms were obtained from subjects or their legal guardians in the case of children. Assent forms were obtained directly from children. The experiment was conducted on a group of 22 male subjects, 5 nonasthmatic adults, 8 nonasthmatic children, and 9 clinically diagnosed asthmatic children. Non-smoking adults between 25 and 35 years of age with no history of asthmatic symptoms were recruited at the University of Texas at El Paso. Children between the ages of 9 and 16, from nonsmoking households, were randomly selected from an existent cohort of 500 children. The cohort was built for purposes of a larger epidemiological study for which children asthmatic status was confirmed as described next. Suspected asthmatics were identified based on the standardized asthmatic symptom prevalence questionnaire from the International Study of Asthma and Allergies in Childhood (ISAAC) [49]. Subsequently, lung function tests (spirometry and bronchodilator response) were performed following the American Thoracic Guidelines (ATS) [50]. A forced expiratory volume in 1 s (FEV_1_) ≥ 90% of predicted was used as a healthy threshold. A forced expiratory volume in 1 s (FEV_1_) ≤ 80% of predicted, a ratio of FEV_1_ to forced vital capacity (FVC) ≤ 75%, and a positive bronchodilator response were used as asthmatic thresholds. A positive bronchodilator response was defined as an increase in FEV_1_ ≥ 15% and/or ≥200mL from baseline after inhalation of 400 *μ*g of albuterol. 

### 2.2. Experimental Design

 The experiment was conducted between February and May, 2010. Deposition measurements were conducted during uncontrolled breathing. Breathing frequency (breaths per minute, bpm), tidal volume (liters, *L*), and minute ventilation (liters per minute, lpm) were recorded with a pneumotachograph (PNT) during exposure. Height, weight, and body mass index (BMI) were also documented. The variables were categorized into groups representing varying ventilation and physiological conditions. The ventilation group included breathing frequency, minute ventilation, and tidal volume, whereas the physiological group included BMI, height, weight, and age. The objective was to evaluate the combined influence of varying ventilation and physiology on UFP deposition, by paring and controlling for variables from these two groups. Pulmonary function immediately before exposure was assessed by means of forced expiratory volume and forced vital capacity measured with an EasyOne spirometer (NDD Medical Technologies, Andover, MA) following previously documented protocols [51]. Pearson correlation was employed to assess associations between variables. Deposition means between groups were compared by two-tailed Student's *t*-tests with *P* < 0.05 denoting significance [52]. The effect of asthmatic condition adjusted for ventilation and physiological variability was explored via one-way analyses of covariance (ANCOVA) [53]. The International Commission on Radiological Protection (ICRP 66) deposition model [48] was employed to assess the effect of varying ventilation independently.

### 2.3. Exposure

Subjects were exposed to polydisperse sodium chloride (NaCl) produced via atomization (TSI. Inc., Model 3076) of a salt-deionized water solution of 1% by mass. The NaCl aerosol had a geometric mean mobility diameter (GMD) of 27–31 nm, a geometric standard deviation (GSD) of 1.8–2.0, and a total concentration of 1.2 × 10^6^ particles cm^−3^. The particle number concentration is comparable to the levels of ultrafine particle typically observed near dense traffic highways [54–56]. Sodium chloride particles were used because they do not exacerbate asthmatic symptoms. The system was extensively characterized with NaCl particles and particle shift and loss were known to be minimal [57]. Two size-resolved deposited fraction (DF) curves for particles with mobility diameters in the range 10–200 nm were determined per subject per measurement. Each measurement was duplicated on a nonconsecutive day. Each DF curve measurement was obtained during a 12-minute exposure period. A short period was desired to facilitate children's participation. The exposure period was defined as the shortest time span for which consistent measurements were achieved. Exposure was through a silicone mouthpiece assisted by a nose-clip. The use of a mouthpiece has been observed to affect breathing patterns by increasing minute ventilations during respiratory measurements [58]. The instrumentation employed to measure the ultrafine particle concentrations requires the capture and retention of uncontaminated exhaled breath samples; thus the use of a mouthpiece was necessary. By controlling for ventilation in the ANCOVA mouthpiece-induced breathing variability was also accounted for. 

### 2.4. Deposition Measurements

**Figure 1:**
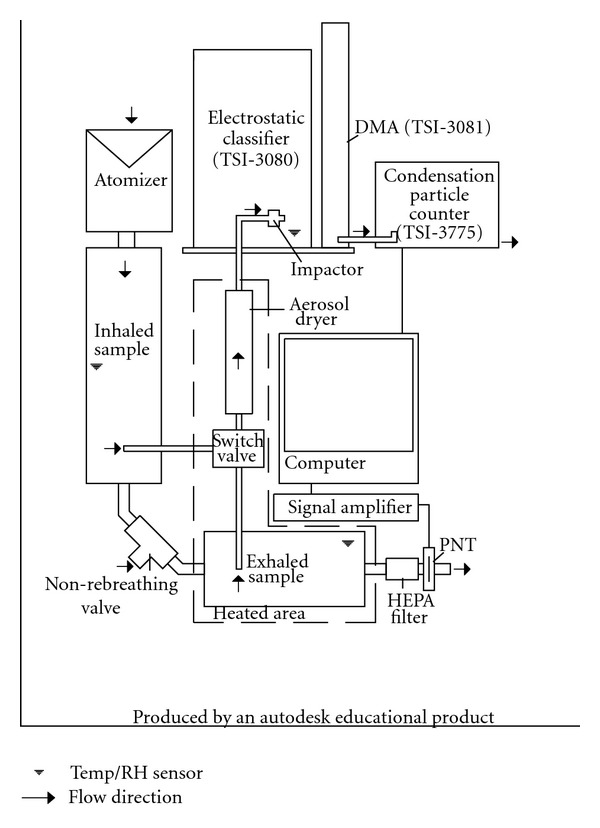
Layout of flow-through breath sampling system.

A scanning mobility particle sizer (SMPS 3936-L75, TSI Inc. USA) was employed to determine particle size distributions and number concentrations in breath samples. Breath samples were delivered to the SMPS via a well-characterized flow-through system [57]. To accommodate children's breathing conditions and further reduce particle loss, a custom-made aluminum non-rebreathing valve and a smaller exhaled sample tank (2.5 L) were introduced to the system originally presented by Löndahl et al. [57]. The non-rebreathing valve had a dead space of 19.5 cm^3^. The temperature of the exhaled sample was maintained at 37°C until dried to prevent condensation and minimize size shift due to coagulation. To avoid the effects of temperature on concentration due to air volume changes, inhale and exhale samples were dried and cooled to identical conditions before reaching the SMPS. The system operated at ambient pressure. A diagram of the flow-through system is shown in Figure 1.

The inherent particle loss in the modified system was measured as in Löndahl et al. [57] and was reduced to 5% as compared to the 10% previously reported. The highest size-dependent particle loss was below 5 ± 0.8% and occurred at the smallest measured particle size (5.9 nm) and it decreased with particle size. Measurements for particles smaller than 10 nm were discarded. Deposition fractions were estimated as follows:

(1) DFhuman(dp,i)=1−Cex(dp,i)Cin(dp,i)·(1−DFequip(dp,i)),

where *d*_*p*,*i*_ is the particle diameter in size channel *i*, *C*_in_ and *C*_ex_ are the particle concentrations in the inhaled and exhaled samples, respectively, and DF_equip_ is the particle loss incurred in the system. The equation is valid for depositional losses occurring in any part of the system between the two sampling ports providing that the particles do not change size during measurements. The SMPS produced DF curves consisting of 99 logarithmically spaced size bins within a mobility diameter size range of 10–200 nm. To further minimize the effect of particle size shift on the DF measurements, the number of size-bins was reduced to 33 by increasing the size range of the bins. Total deposited fraction (TDF) of the NaCl aerosol in the particle size range of 10 nm–225 nm was calculated as in the equation by using the total number concentration for the inhaled and exhaled breath samples.

### 2.5. Model

Deposition in the respiratory tract of healthy subjects was estimated with the empirical ICRP 66 model [48]. The ICRP 66 was selected for this study as it has been shown to produce comparable results to most deposition models and has been widely referenced in similar studies [47, 57]. The anatomical and physiological reference values for 15- and 10-year-olds provided by the ICRP 66 model were employed in this study. Hygroscopic growth was estimated under the assumption of RH = 99.5% throughout the respiratory tract and immediate particle growth to the equilibrium size [48]. Deposition estimates produced with ICRP 66 model were made for an aerosol with the same characteristics as the one used during the experiments, that is, with a GMD of 30 nm, GSD of 2.0, and a total concentration of 1.2 × 10^6^ particles cm^−3^. 

## 3. Results

### 3.1. Child versus Adult

**Figure 2:**
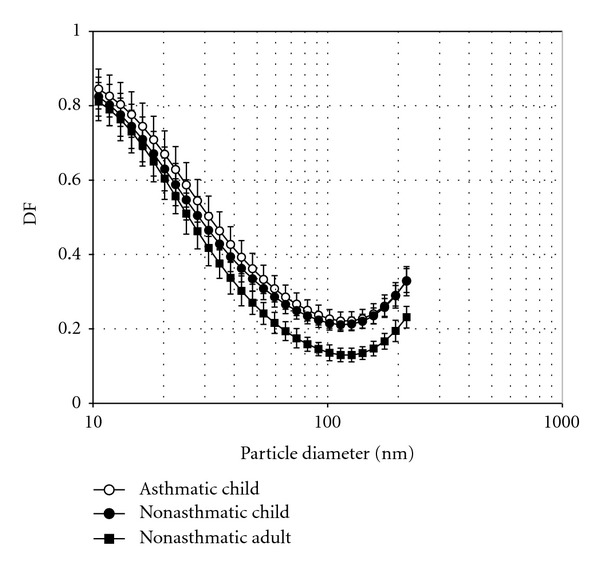
Measured deposition fraction curves for all subjects. Error bars represent 95% confidence intervals. The DF curves show a significant difference between children and adults and a nonsignificant difference between asthmatic and non-asthmatic children.

The dataset is presented in Table 1 and mean and standard deviations of age, sex, BMI, respiratory parameters, and TDF by subject group are summarized in Table 2. The average BMI percentiles by age for all three subject-groups were below the 85 percentile overweight criteria [59]. However, individually six children had a BMI in the overweight percentile range and two in the obese percentile range [59]. As expected, children had higher breathing frequencies and lower tidal volume and minute ventilation than adults [60, 61]. During the uncontrolled breathing measurements TDF for both asthmatic and non-asthmatic children was higher as compared to healthy adults. Specifically, non-asthmatic children had 50% higher TDF than non-asthmatic adults (*P* = 0.002) for ultrafine hygroscopic particles with dry mobility diameters of 10 to 200 nm (see Table 2). The curves in Figure 2 show that the significant differences in size resolved DF between adults and children, occurred for diameters greater than 50 nm.

### 3.2. Asthmatic versus Non-Asthmatic

**Figure 3:**
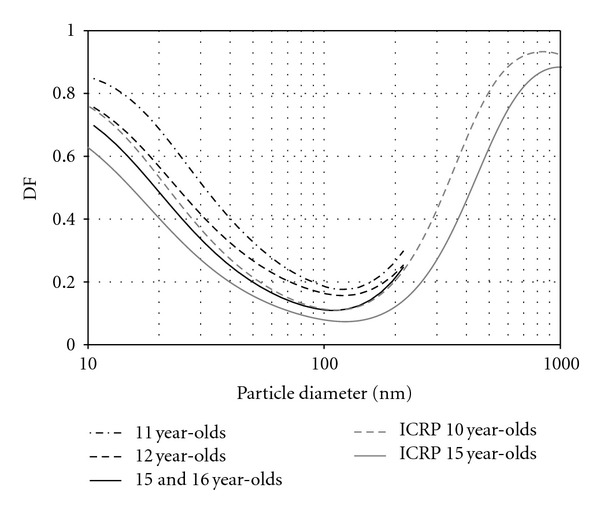
Measured and modeled deposition fraction curves by age for non-asthmatics. The curves suggest a strong effect of age on deposition. Age was used as a covariate in the ANCOVA analysis for total deposited fraction. Note reasonable agreement between our measurements and the ICRP model estimates.

The asthmatic group experienced a decrease of minute volume (*V*_*E*_) and breathing rate (*f*) as compared to non-asthmatic subjects. The mean TDF was 14% higher for asthmatic children as compared to non-asthmatics (see Table 3). However, the TDF difference among asthmatic and non-asthmatic children was not significant (*P* = 0.212). The effect of the wide variation of breathing patterns and age within and among the two subjects groups on TDF is further explored in the following sections. As with TDF, the size-dependent DF curves did not suggest a significant difference between the asthmatic and nonasthmatics as shown in Figure 2. The modeled DF curves for healthy 10 and 15 year-olds slightly underestimated the deposition in healthy children, but still performed acceptably well (see Figure 3). Mean *V*_*t*_ and *f* values shown in Table 1 for asthmatic and non-asthmatic children were employed in the ICRP model to estimate TDF for the two groups. Based solely on varying breathing conditions, the ICRP model predicted a positive effect on TDF of 16% and 5% for an “asthmatic” 15-year-old and 10-year-old child, respectively (see Table 3). An age-related effect was also evident from the size-resolved DF curves shown in Figure 3.

### 3.3. Correlation Analysis

Pearson correlation coefficients for observations from the entire group and for children only are shown in Table 4. For the entire group, significant correlations were observed between TDF and BMI, *V*_*T*_, and *V*_*E*_. Age significantly correlated with weight, height, BMI, and *f*. Whereas BMI was inversely correlated with TDF and *f* and directly correlated to *V*_*T*_ and *V*_*E*_. Among children, TDF was only significantly correlated to *V*_*E*_. Correlations between age and *V*_*T*_, and BMI and *f* were not significant among children. These two pairs of variables, each representing ventilation and a physiological characteristic, were used as covariates to evaluate the effect of asthmatic conditions among children.

### 3.4. Adjusted Effects

A series of evaluations of the effect of asthmatic condition on TDF while controlling for age, BMI, height, weight, *f*, *V*_*T*_ and *V*_*E*_ independently did not reveal significant results. However, after controlling for age and *V*_*T*_ or BMI and *f*, significant effects on TDF due to asthmatic conditions were observed (see Tables 5 and 6). The age-*V*_*T*_ ANCOVA produced the most significant results; *F*(1, 13) = 7.419, *P* < .05 and 20.4% (*w* ^2^ = 0.204) of the total variance in TDF was accounted for by the two levels of asthmatic condition controlling for the effect of subject age and tidal volume during the experiment. The adjusted TDF mean for asthmatics (0.552) was 21% higher than for non-asthmatics (0.458) for an age value of 12.06 years and *V*_*t*_ of 0.37 L (see Table 3). The homogeneity-of-regression (slopes) assumption was confirmed as the relationship between the covariates, and the dependent variable did not differ significantly as a function of the independent variable: *F*(1, 10) = 1.195, *P* = .300 for tidal volume and *F*(1, 10) = .176, *P* = .684 for age. 

## 4. Discussion

Bennett and Zeman [58] established that for identical DF values, and independent of particle size, the deposition rate is higher in children than in adults due to higher minute ventilation and smaller lung surface area. Bennett and Zeman [58] also observed that the normalized deposition rate for monodisperse 2 *μ*m particles was actually 35% higher in children as compared to adults. In this study, it was observed that for UFPs, specifically for hygroscopic particles with mobility diameters between 10 and 200 nm, the total deposited fraction was 50% higher in children as compared to adults (see Table 2). Following the approach presented by Bennett and Zeman [58] and using the same functional residual capacity values for adults (2.87 L) and 12 year olds (1.77 L), the 50% increase in TDF for UFP observed in this study results in a normalized deposition rate 32% higher in healthy children as compared to adults. These results suggest that children are prone to considerably higher dosages of airborne ultrafine particles as compared to adults and that such disposition increases with smaller particle size. 

The mean differences in TDF between asthmatic and non-asthmatic children were nonsignificant and strongly affected by age and breathing patterns. For ultrafine hydrophobic particles, Chalupa et al. [47] and Diagle et al. [62] observed significantly higher (42.6%) deposition fractions in asthmatic adults as compared to non-asthmatic adults. However, the effect on total particle deposition variation induced by distinctive breathing patterns among subject groups observed in those studies was undetermined. In this study, after controlling for extraneous variance associated with age and *V*_*T*_ or BMI and *f* a significant effect due to asthmatic conditions was also observed for children. For children, the estimated adjusted effect of asthmatic condition on TDF was 21%. The ICRP model, based solely on varying breathing conditions between the asthmatic and non-asthmatic groups, predicted an effect between 5% and 16%. The model results suggest that a considerable fraction of the increase in DF in asthmatics is directly associated with the variation of *f*, and *V*_*T*_ induced by asthmatic conditions. Enhance diffusional deposition of UFPs might also be associated with airway obstruction and increased lung residual volume, characteristic of asthmatic conditions. In this regard, the disposition to higher dose in asthmatic children might be linked to inflammatory airway conditions, higher minute ventilation, and a smaller lung surface area. The results of this study, which build upon and complement a series of previous studies, suggest that children, and evermore so asthmatic children, receive significantly higher dosages of ultrafine particles than adults with similar exposure.

This study, as well as other deposition studies [47, 63, 64], worked with a small sample size due to constrains of the experimental procedure. The results are preliminary, not only because the subject group is small but also because the breathing conditions were through a mouthpiece and therefore might be unrepresentative of “real” breathing conditions. Still the results clearly substantiate the need for more comprehensive studies on the mechanisms and sources of variability in nanosize particle deposition within the human respiratory tract. Specifically, studies with controlled breathing conditions on asthmatic children, with hydrophobic particles, and on larger groups are necessary. Additional exercises to determine if the accuracy of the ICRP in determining the effect of DF in asthmatic children could be improved by employing subject-specific biometric values would also be valuable. Given the high prevalence of childhood asthma and the potentially higher susceptibility of asthmatic children to air quality impacts, the results of this study were deemed important.

## 5. Conclusion

The results of this study suggest that for the same exposure, children receive a higher lung dose of UFP as compared to adults and that asthmatic condition further increases deposited fraction. The total deposited fraction and normalized deposition rate were 50% and 32% higher in children than in adults, respectively. After controlling for age and tidal volume variation, TDF was 21% higher in asthmatic than in non-asthmatic children. The effect on TDF of distinctive tidal volume and breathing frequency induced by asthmatic conditions was estimated by the ICRP to be between 16% and 5%, for a 10 to 15 year old age range. The results suggest that the observed higher deposited fraction of UFP in asthmatics is due to causes beyond distinctive breathing patterns and possibly due to diffusional deposition enhanced by inflamed airways. The higher health risk of air pollution exposure commonly observed in children, and asthmatics might be linked to higher dosages of UFP, as compared to adults and healthy individuals. 

The subject group studied was small, composed of only males, and the breathing conditions were through a mouthpiece, and therefore might be unrepresentative of “real” breathing conditions. Given the high prevalence of asthma among children the suggested susceptibility to UFP levels is of importance and warrants further research.

## Figures and Tables

**Table 1:** Measurements by participating subject.

Subject	Condition	Sex	Age	Weight (kg)	Height (cm)	Chest width (cm)	BMI	BMI %ile	*f* (bpm)	*V*_*t*_ (L)	*V*_*E*_ (L/min)	TDF
A	Asthmatic	M	9	33.8	137	76	18.1	80	24.0 ± 1.41	.14 ± .01	3.24 ± .02	.49 ± .00
B	Asthmatic	M	9	36.6	142	77	18.2	81	16.0 ± .00	.26 ± .01	4.08 ± .11	.46 ± .03
C	Asthmatic	M	10	36.3	145	71	17.4	63	22.0 ± 5.66	.16 ± .05	3.27 ± .21	.61 ± .05
D	Asthmatic	M	10	42.0	147	76	19.4	85	18.5 ± .71	.23 ± .02	4.17 ± .55	.60 ± .01
E	Asthmatic	M	12	56.8	162	82	23.5	89	16.8 ± .32	.54 ± .04	9.07 ± .27	.51 ± .03
F	Asthmatic	M	12	56.4	150	81	25.1	96	13.7 ± .00	.66 ± .01	9.04 ± .19	.43 ± .01
G	Asthmatic	M	15	56.6	167	77	20.3	58	9.5 ± 2.12	.37 ± .13	3.38 ± .42	.64 ± .01
H	Asthmatic	M	16	63.5	174	80	21.0	56	7.4 ± .92	.48 ± .01	3.52 ± .34	.65 ± .01
I	Asthmatic	M	12	54.3	158	80	20.5	78	15.2 ± .32	.61 ± .04	9.70 ± .50	.43 ± .01
J	Nonasthmatic	M	11	53.1	160	77	20.8	88	31.5 ± 2.12	.20 ± .05	6.20 ± 1.97	.42 ± .06
K	Nonasthmatic	M	11	45.3	152	70	19.5	80	39.5 ± .71	.17 ± .01	6.72 ± .68	.61 ± .06
L	Nonasthmatic	M	11	40.8	142	74	20.2	85	16.5 ± .46	.61 ± .01	9.98 ± .39	.37 ± .00
M	Nonasthmatic	M	12	61.2	170	84	21.2	86	19.0 ± .00	.24 ± .02	4.47 ± .40	.53 ± .02
N	Nonasthmatic	M	12	57.1	168	76	20.4	81	23.5 ± 2.12	.15 ± .01	3.51 ± .01	.36 ± .04
O	Nonasthmatic	M	12	52.2	155	84	21.8	88	15.2 ± .28	.52 ± .02	7.81 ± .46	.42 ± .01
P	Nonasthmatic	M	15	54.2	170	76	18.9	34	12.5 ± 2.12	.47 ± .15	5.66 ± .87	.55 ± .07
Q	Nonasthmatic	M	16	80.3	176	90	26.2	92	12.5 ± 2.12	.51 ± .12	6.19 ± .43	.55 ± .07
R	Adult	M	21	74.5	174	85	24.6	—	12.9 ± .92	.64 ± .11	8.26 ± .55	.36 ± .06
S	Adult	M	36	86.6	180	91	26.6	—	12.1 ± 1.10	.68 ± .06	8.23 ± .24	.38 ± .00
T	Adult	M	22	78.3	175	84	25.6	—	13.4 ± .67	.62 ± .11	8.31 ± .46	.34 ± .05
U	Adult	M	20	69.4	171	71	23.7	—	16.7 ± .85	.54 ± .09	9.02 ± .76	.30 ± .04
V	Adult	M	29	88.7	181	88	25.8	—	10.00 ± .89	.82 ± .07	9.12 ± .39	.42 ± .02

^∗^ Mean values ± standard deviation for four experimental repetitions.

**Table 2:** Subject demographics and summarized results^∗^.

Characteristic	Asthmatic (FEV1% < 80)	Nonasthmatic^∗∗^ (FEV1% > 90)	Total children	Adult (control) (FEV1% > 90)
Age (years)	11.67 ± 2.50	12.50 ± 1.93	12.06 ± 2.22	25.60 ± 7.16
Weight (kg)	48.52 ± 11.20	51.79 ± 11.88	51.79 ± 11.74	79.5 ± 8.12
Height (cm)	153.6 ± 12.38	157.35 ± 11.41	157.35 ± 12.28	176.11 ± 4.08
BMI	20.39 ± 2.55	20.74 ± 2.25	20.74 ± 2.37	25.26 ± 1.12
BMI-Percentile	76.22 ± 14.09	77.65 ± 18.69	77.65 ± 15.95	— ± —
*f* (bpm)	15.98 ± 5.34	18.47 ± 9.71	18.47 ± 7.93	13.01 ± 2.45
*V*_*t*_ (L)	.38 ± .20	.37 ± .18	.37 ± .19	.66 ± .34
*V*_*E*_ (L/min)	5.50 ± 2.85	6.31 ± 1.99	5.88 ± 2.44	8.59 ± .44
TDF^∗∗∗^	.54 ± .09	.48 ± .10	.51 ± .09	.36 ± .05

^∗^ Mean values ± standard deviation.

^∗∗^ Including a passive smoker.

^∗∗∗^ Total deposition fraction for an aerosol with GMD of 40 nm, *σ*_*g*_ of 1.9, and mobility diameter range from 10 to 200 nm.

**Table 3:** Summary of total deposited fraction values.

Characteristic	Asthmatic	Nonasthmatic	Effect	Notes
Mean	.544	.476	14%	All subjects
Adjusted Means	.552	.458	21%	ANCOVA
ICRP for 15 year olds^∗^	.507	.438	16%	Based on distinct *f* and *V*_*t*_ values as shown in Table 2
ICRP for 10 year olds^∗^	.501	.475	5%	Based on distinct *f* and *V*_*t*_ values as shown in Table 5

^∗^ Estimated for an aerosol of GMD = 30 nm, *σ*_*g*_ 2.0, and total concentration 1.2 × 10^6^ particles cm^−3^.

**Table 4:** Pearson correlation coefficients for variables.

		Age	Weight	Height	BMI	*f*	*V*_*T*_	*V*_*E*_	TDF
		Only children							
Age		**1.000**	.836^∗∗^	.867^∗∗^	.507^∗^	−.571^∗^	0.478	0.004	0.353
Weight		.865^∗∗^	**1.000**	.895^∗∗^	.794^∗∗^	−0.389	0.427	0.143	0.091
Height		.757^∗∗^	.922^∗∗^	**1.000**	0.473	−0.337	0.208	−0.102	0.247
BMI	All Subjects	.743^∗∗^	.896^∗∗^	.692^∗∗^	**1.000**	−0.313	.617^∗∗^	.494^∗^
*f*		−.459^∗^	−.493^∗^	−.455^∗^	−.441^∗^	**1.000**	−.649^∗∗^	−0.031	−0.123
*V*_*T*_		.668^∗∗^	.692^∗∗^	.509^∗^	.768^∗∗^	−.688^∗∗^	**1.000**	.753^∗∗^	−0.219
*V*_*E*_		0.402	.432^∗^	0.215	.627^∗∗^	−0.174	.798^∗∗^	**1.000**	−.503^∗^
TDF		−0.385	−0.365	−0.187	−.477^∗^	0.075	−.454^∗^	−.629^∗∗^	**1.000**

^∗∗^ Correlation is significant at the 0.01 level (2-tailed).

^∗^ Correlation is significant at the 0.05 level (2-tailed).

**Table 5:** Analysis of covariance for total deposited fraction by asthmatic condition.

Source	SS	df	MS	*F*	*P*
Age	0.057	1	0.057	12.199	0.004
Tidal volume	0.04	1	0.04	8.578	0.012
Asthmatic condition	0.035	1	0.035	7.419	0.017
Error	0.061	13	0.005		

Total	0.142	16			

**Table 6:** Analysis of covariance for total deposited fraction by asthmatic condition.

Source	SS	df	MS	*F*	*P*
BMI	0.047	1	0.047	7.778	0.015
*f*	0.022	1	0.022	3.608	0.080
Asthmatic condition	0.043	1	0.043	7.154	0.019
Error	0.081	13	0.006		

Total	0.140	16			
